# A review on the microbiology of Ethiopian traditional fermented beverage products

**DOI:** 10.3389/fnut.2025.1519547

**Published:** 2025-01-29

**Authors:** Guesh Mulaw, Trhas Gebregziabher, Teklemichael Tesfay

**Affiliations:** Department of Biology, College of Natural and Computational Sciences, Aksum University, Aksum, Ethiopia

**Keywords:** bioavailability, fermentation, nutritional value, traditional beverage, Ethiopia

## Abstract

Traditional fermented beverages are drinks produced locally on the basis of ethnic knowledge and consumed nearby the locality of production. Ethiopia is a country where a wide variety of traditional fermented beverages are prepared and consumed. *Tella*, *borde*, *shamita*, *korefe*, *cheka*, *tej*, *booka*, *grawa*, *areki*, and *keribo* are among the traditional fermented beverages in Ethiopia. This review paper highlights the fermentation process and nutritional value of traditional fermented beverages, microorganisms involved in the traditionally ferreted beverages, the nutritional value and shelf-life of fermented beverages, as well as the bioavailability and safety by collecting recent research articles. These traditional fermented beverages significantly enhance health due to the presence of bioactive compounds and their nutritional value relatively greater than those of nonfermented beverages. The fermentation byproducts of yeast and Lactic Acid Bacteria (LAB) increase the acidity of beverages and are crucial for maintaining the quality and characteristics of fermented beverages. It also helps to reduce the amount of toxins and pathogens in food. Similarly, fermented foods contain probiotics, which are beneficial bacteria that help the body to digest food and absorb nutrients. The fermented foods and beverages are important in preventing non-communicable diseases such as cardiovascular diseases, gastrointestinal tissues, immune disorders, and cancer. Overall, the paper provides a comprehensive overview of the current knowledge and tradition on Ethiopian fermented beverages.

## 1 Introduction

Fermentation is one of the oldest methods of preservation, and fermented beverages have been known for their nutritional value since prehistoric times (1). Traditional fermented beverages are culturally and socially acknowledged products for consumption, drinking, entertainment, customary practices, and religious purposes (2). The functions of microorganisms have been linked to several health benefits; for example, fermented beverages have been shown to improve immunity and control digestive disorders, cardiovascular disease, and other health issues. Microbes such as bacteria (LAB), fungi, and molds are the main reasons for these health benefits (1).

Traditionally fermented beverages have been fermented to improve the shelf-life of the products, unlike these microorganisms, which are produced through acidification, alcoholization, proteolysis, and/or amino acid conversion, to produce products with desirable quality characteristics in terms of shelf life, texture, taste, flavor and color (3). These aspects make traditional fermented beverages not only healthy but also available on the commercial market (1). The methods for preparing these traditional beverages have passed down from generation to generation (4).

In many cases, the nutritional value and shelf life of non-fermented beverages are relatively lower than those of fermented beverages. Fermented beverages are highly important in society, with a high protein contribution, as they involve microbial processes that benefit human health (5). Fermented beverages contain bioactive substances that can help reduce or inhibit allergies and diseases. Fermentation, as a key technique for producing high-quality food products, plays a crucial role in enhancing beverages by preserving sensory and nutritional qualities such as protein and vitamins (6).

In traditional fermented beverages, the source of microorganisms involved in fermentation processes predominantly originates from the microflora naturally present in the substrates and the utensils/equipment used for fermentation. During fermentation, the microbial groups coexist to enhance fermentation processes by adapting to the changing intrinsic and extrinsic conditions caused by the physicochemical changes associated with microbial activity, duration of fermentation, temperature, and moisture content (7).

Many varieties of fermented beverages have been studied and analyzed worldwide, and new additions have been made to make them commercially viable (1). Asia is well known for its exotic, traditionally fermented food and beverage products produced from a wide range of raw materials, microorganisms, and fermentation processes (8). In several African countries, traditional fermentation processes provide a means of preserving food, improving shelf-life, and adding nutrients to food products (9). In developing countries, fermentation is a traditional food processing method for the production of relatively safe and nutritious foods (10).

Ethiopia is a country where a wide variety of traditional fermented beverages are produced and consumed for a long period of time. Traditional fermented beverages are those that are indigenous to a particular area and have been developed by people from locally available raw materials via age-old techniques (11). This study sought to review the available information on the processing techniques involved in the production of these beverages, the species of various microorganisms involved in fermentation processes and the nutritional value of these traditional fermented beverages in Ethiopia (Table 1).

**Table 1:** Microbes associated with the fermentation of different Ethiopian fermented beverage product.

Beverages	Raw materials	Microbes involved	PH	Alcohol content	Consumption place	References
*Tella*	*Gesho*, barley, wheat, maize, millet, sorghum and teff	*Saccharomyces cerevisiae*, *Lactobacillus* spp., *Acetobacter* spp. and *Bacillus* spp	4–5	4–6% (v/v)	Amhara Tigray, Oromia, Addis Ababa and SNNP	(95)
*Areki*	*Gesho*, barley, sorghum, maize and teff	LAB and yeast	4.3	38–48% (v/v)	Amhara, Tigray, Oromia, Addis Ababa, SNNP	(8)
*Borde*	malt, maize, barley and wheat	AMB, Staphylococci, ASFB, LAB Enterobacteriaceae, and yeast	3.8	3.35 ± 0.64 (% v/v)	South nation nationality of peoples (SNNP)	(39)
*Shamita*	Barley, maize and wheat	LAB and yeast	4.03	3.2% v/v	SNNP, Addis Ababa	(27)
*Keribo*	Barley, honey and sugar	AMB, ASF, LAB and yeast	4.2	4.5% v/v	Amhara, Oromia, Addis Ababa	(36)
*Korefe*	Barley and *gesho*	Lactococci, Lactobacilli*, Entrobacteriaceae* and yeast	4	4.08–5.44% v/v	Amhara (Koumant ethnic group)	(12)
*Tej*	Honey and *Gesho*	AMB, Staphylococci, *coliforms*, Enterobacteriaceae, LAB and yeast	3.81	7–11% v/v	Amhara, Tigray, Addis Ababa	(60)
*Booka*	Bladder of cow and honey	*E. coli, Enterobacteriaceae*, *Lacobacillus* and *coliforms*	3.01	1.53% (v/v)	South Ethiopia, *Guji* community	(13)
*Cheka*	Sorghum, maize, finger millet, vegetables and root of taro	Yeast and LAB	4.11	4.05–6.75% (v/v)	SNNP	(11)
*Grawa*	Honey (made from flower of *Vernonia amygladina*)	AMB, ASFB, LAB, Staphylococci, *Enterobacteriaceae, coliforms*, yeast	3.6	5.3% v/v	Anfilo District, Wollega Zone, Southwest Ethiopia	(7)

## 2 Traditional fermented beverages in Ethiopia

Traditional fermented beverages are drinks produced locally using indigenous knowledge and consumed near the vicinity of production. In Ethiopia, the preparation and consumption of cereal- and fruit-based traditional fermented beverages are very common (12). Indigenous processing methods for fermented Ethiopian beverages differ from place to place or from product to product. Among the Indigenous fermented beverages in Ethiopia, *tej*, *tella*, and *areki* are considered alcoholic, whereas *cheka*, *korefe*, *shamita*, *keribo*, *borde*, and *booka* are considered nonalcoholic drinks. The fermentation of traditional Ethiopian beverages is spontaneous, natural and uncontrolled (11). Low-alcoholic nutritional value Ethiopian beverages have greater nutritional value. Thus, they can be used as food replacements. These traditional alcoholic beverages also contain a significant amount of total polyphenols and antioxidants (12). The alcohol content and pH values of these beverages range from 1.53–21.7% and 2.9–4.9, respectively (13).

The source of microorganisms responsible for fermentation is mainly the ingredients and utensils. These traditional alcoholic beverages are of variable quality within and between products. This is due to the high number of live cells present in freshly produced beverages. Yeasts and lactic acid bacteria are the predominant microorganisms encountered during the fermentation of these traditional alcoholic beverages (7).

### 2.1 Tella

*Tella* is a ubiquitous and popular beverage in Ethiopia, often called traditional Ethiopian beer (14). *Tella* is a fermented traditional beverage with a color ranging from grayish-white to brown, depending on the degree of roasting, and it is the most widely brewed and consumed alcoholic beverage in almost every household (12, 15). The intensity of specific processing steps determines the color of a beverage during preparation. *Tella* is prepared in Ethiopia from cereals such as barley, wheat, maize, millet, sorghum, and teff.

#### 2.1.1 Preparation of *Tella* fermentation

The *tella-*making process and its raw materials vary among ethnic groups and among economic and traditional situations (16). Although there are minor changes in the process in different localities, the basic steps are similar throughout the country. The making of *tejets, tenses* and *difdifs* is the fundamental step in the *tella* preparation process (17). The *tella-*making process starts by soaking the barley in water for approximately 24 h at room temperature to produce malt, which is locally called *bikil*. After 24 h, the moistened grain was covered with fresh banana leaves and kept in a dry place for an additional three days (18). The germinated barley grain was sun-dried and ground to produce malt flour. At the same time, *gesho* (*Rhamnus prinoides*) leaves and stems are sun-dried and ground. Then, the *bikil* flour and *gesho* powder are mixed with an adequate amount of water in a clean and smoked traditional bioreactor known as an *insera*.

This mixture is left to ferment for two days to form *a tejet*. The millet, sorghum and teff (*Eragrostis tef*) flours in equal proportions were subsequently mixed with water to form a dough. The dough is then baked to produce unleavened bread locally known as *ye tella kita*, which is sliced into pieces and added to the earlier, produced *tejet*. The mixture is then sealed tightly to ferment anaerobically for 5 to 7 days to turn into *tenses*. While the *tenses* ferment, the maize grain is soaked in water for approximately 3 days and then dried, roasted and ground to make dark maize flour called *asharo*. *Asharo* is the main ingredient that determines the color of the *tella* (19). *Asharo* is then added to the previously produced *tenses* and fermented anaerobically for a period of 10--20 days. After this period of fermentation, a thick mixture locally called *difdif is* formed. Water is added to *difdif* and left to ferment for an additional 5 to 6 h. Finally, solid residues are removed by filtration and serve to consumers as *tella*. To produce 25 to 28 L of pure *tella*, 1 kg of *gesho* (*R. prinoides*) powder, 0.5 kg of *bikil*, 5 kg of *ye tella*, 10 kg of *asharo* and 30 L of water are needed (20).

**Figure 1:**
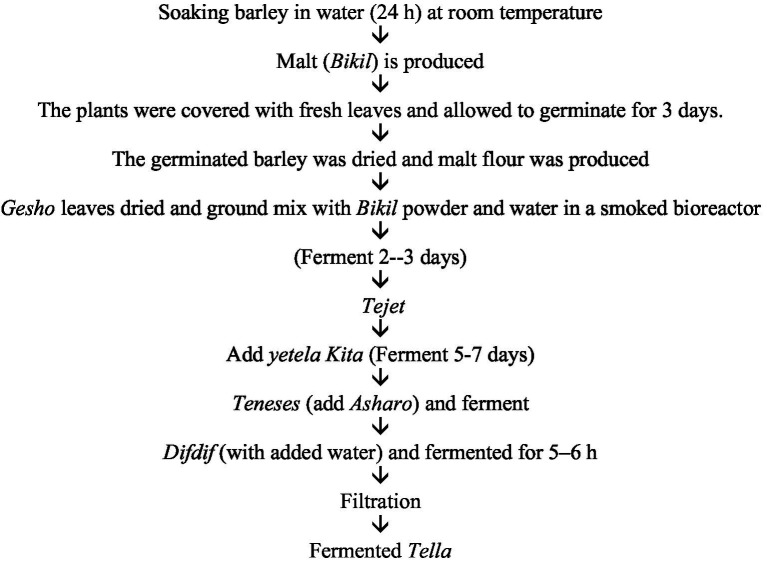
Flow chart of *Tella* fermentation.

The *grawa* (*Vernonia amygdalina*), also known as the bitter leaf, is used as a cleaning agent for *tella* containers and has medicinal properties. Additionally, weira (*Olea europaea*), a subspecies of olive, contains various chemicals that act as a multii-chemical defense against insect and microbial attacks. In Ethiopia, weira is commonly used for smoke fermentation in traditional drink containers, including those for *tella*, water, milk, and milk products. After the container is cleaned and prepared, it is inverted over smoking wood fragments of weira for 10–15 min, removing microorganisms sensitive to wood smoke and adding flavor to *tella* (8). Weyra provides flavor for *tella*, can kill bacteria, and extends *tella* shelf life. The quality of *tella* depends on the quality and safety of the raw materials used and the method of preparation. *Tella* typically has a shelf life of 5–7 days at room temperature (17). In addition to being used in some parts of the country, the leaf of *Croton macrostachyus* is used for cleaning, and dry *Aloe vera* is used for smoking the equipment used for *tella* fermentation (Figure 1).

#### 2.1.2 Microbiology of *Tella* fermentation

The ingredients and utensils used to prepare *tella* are the major sources of microorganisms for fermentation. The genera Saccharomyces, Lactobacillus and Acetobacter are the predominant fermenting microorganisms present in *tella* (17). The fermenting organisms were composed of Saccharomyces spp. (mostly *S. cerevisiae*) and *Lactobacillus* spp. (mostly *Lactobacillus pastorianumi*). The yeasts dominated the fermenting flora after the end of the first stage until the completion of fermentation (21).

The microbial population of a control *tella* sample on the first day after its preparation, which contained yeast and some bacterial species. The control *tella* on the 14th day contained even more yeast and bacteria. The number of colonies found in the pasteurized *tella* samples was 1 × 10^2^ CFU/mL, and the number of colonies found in the vacuum-filtered *tella* samples was 3 × 10^3^ CFU/mL on the 14th day of observation. The vacuum filtration and pasteurization processes dramatically decreased the number of bacterial colonies in the *tella* samples. Further investigation of the microbial profile revealed that although there was no yeast inoculation stage during *tella* fermentation, all the *tella* samples collected on the first day contained yeast, indicating the utilization of the natural yeast present on the cereals for fermentation. The control *tella* exhibited a very large microbial flora, which further increased to 1.05 × 10^9^ on the 14th day from 9.86 × 10^5^ on the 1st day after *tella* preparation. Furthermore, *Escherichia coli*, *Staphylococcus aureus*, and *Shigella flexneri* were identified in the control *tella* after the 14th day of preparation (17) (Table 2).

**Table 2:** Distribution of the of the main ingredient Tella’s microflora.

Ingredient	Enterobacteriaceae	Yeasts	Lactobacillus	Lactococcus	Acetic acid bacteria	Aerobic Mesophilic counts
*Gesho*	5.86 ± 0.01	5.55 ± 0.21	0.00 ± 0.00	0.00 ± 0.00	0.00 ± 0.00	5.85 ± 0.91
*Bikil*	5.88 ± 0.11	5.77 ± 0.10	5.52 ± 0.32	5.79 ± 0.00	0.00 ± 0.00	5.97 ± 0.63
*Kita*	0.00 ± 0.00	0.00 ± 0.00	0.00 ± 0.00	0.00 ± 0.00	0.00 ± 0.00	4.8 ± 0.12
*Asharo*	0.00 ± 0.00	0.00 ± 0.00	0.00 ± 0.00	0.00 ± 0.00	0.00 ± 0.00	4.83 ± 0.34

Source: Berza and Wolde (20).

### 2.2 Areki

*Areki* is a traditional distilled alcoholic beverage in Ethiopia (22). It is clear, colorless liquor that is more frequently brewed and consumed by farmers and individuals in semi urban and rural areas than in urban areas (23). *Areki* holds significant cultural importance in Ethiopia, serving as a traditional alcoholic beverage that is deeply intertwined with social rituals and community gatherings and is often consumed during celebrations, family gatherings, communal events and cultural identities. It is often consumed by individuals with alcohol dependence who cannot pay for factory-made alcohol (24).

#### 2.2.1 Preparation of *Areki* fermentation

**Figure 2:**
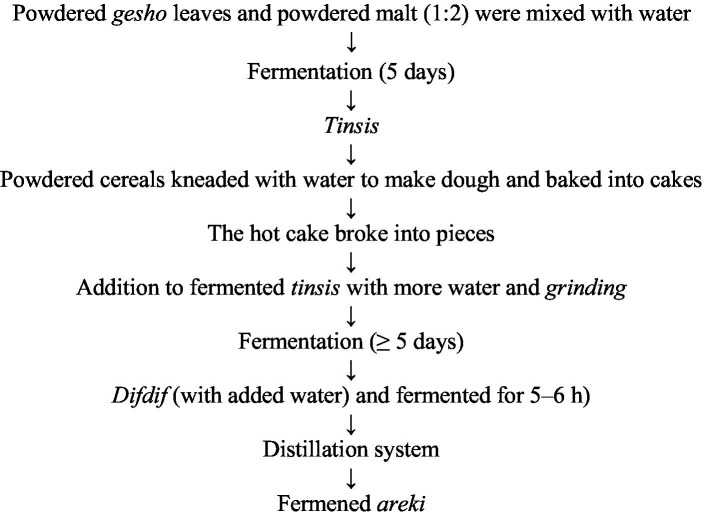
Flow chart of traditional *Areki* preparation.

The production process of *areki* closely resembles that of *tella*, except for a more concentrated fermentation mass and higher alcohol content. The *areki* fermentation product is known as *Yereki-tinsis,* which is prepared by mixing powdered *gesho* leaves and powdered malt in a 1-to-2 ratio with water to form a mixture with a free-flowing consistency that is fermented for approximately five days (11). Then, the powdered dagussa (*Elusine coracann*) is kneaded with water into the dough, baked into cakes, broken into small pieces, mixed well with the first mixture, and allowed to ferment for four days. Then, a part of the mixture is transferred to a traditional distillation material to produce a distilled beverage known as *terra-areki,* which has an alcohol content of 22–34% (v/v). Traditionally, *areki* is classified as *terra-areki* or *dagim-areki*. Terra in Amharic refers to ordinary, an amount of dagussa (*Elusine coracann*) roughly equivalent to four times that of the *bikil* that is powdered. However, *dagim-areki* is a stronger type prepared by redistilling terra-*areki,* resulting in an alcohol content of approximately 45% (v/v). It is prepared in the same way as *terra-areki*, except that the distillation process is allowed to proceed for a shorter period of time, or three volumes of *terra-areki* are redistributed to yield approximately one volume of *dagim-areki*. Finally, the mixture was left to ferment before distillation for 5–6 days. This results in a more concentrated, colorless, and clear traditional alcoholic beverage (18) (Figure 2).

### 2.3 Borde

*Borde* is a cereal-based traditional fermented beverage that is widely consumed in southern and western Ethiopia. It is an opaque, effervescent, whitish-gray to brown-colored beverage with a thick consistency and a sweet–sour taste (7). The *borde* is an important product because both adults and children often consume it as a low-cost meal replacement (7). It is consumed daily, especially during the dry season. An average worker consumes approximately 3 to 5 gourd bottles (1 to 2 litters) of *borde* per day, which would sustain her/him without additional food for most of the day. It is consumed in large quantities at cultural festivals, on market days and at collective work gatherings. The popularity of *bonders* among the population in the region indicates their production in very large volumes. High prices and poor availability of raw materials and cooking fuel during the food-deficient rainy season, from July to September, result in a reduction of the Main Ingredient *Tella’s* microflora or cessation of *borde* production for some vendors (25). Additionally, it is considered to have a low alcoholic libe of the Main Ingredient *tella’s* Microflora Ratio value of 3.35 ± 0.64 (v/v) (26). *Borde* holds special importance for lactating mothers, as its consumption postchildbirth is believed to increase lactation, as highlighted by Kitessa et al. (27).

#### 2.3.1 Preparation of *Borde* fermentation

*Borde* is produced by spontaneous fermentation via simple equipment. It is prepared from various bowls of cereal, such as maize (*Zea mays*), barley (*Hordeum vulgare*), wheat (*Triticum sativum*), finger millet (*Eleusine coracana*), sorghum (*Sorghum bicolor*) and/or tef (*Eragrostis tef*) and their malt, sorghum and tef being the primary ingredients. The major equipment used for the preparation of the *borde* is earthenware pots and griddles, ground stones, bowls, and wonnfit (a sieve with a mesh of interwoven grass-fiber threads at the bottom) (8). The specific ingredients and methods can vary by region, reflecting local preferences and availability (11). The traditional preparation of *borde* involves a series of steps. The first step is soaking; cereal grains (such as maize, barley or millet) are soaked in water for approximately two days to initiate germination. After soaking, the water is drained, and the grains are spread out to germinate for 2–3 days. The germinated grains are then dried and ground into coarse flour. The flour is mixed with water and allowed to ferment for approximately 3–4 days, resulting in a sweet–sour beverage (26).

**Figure 3:**
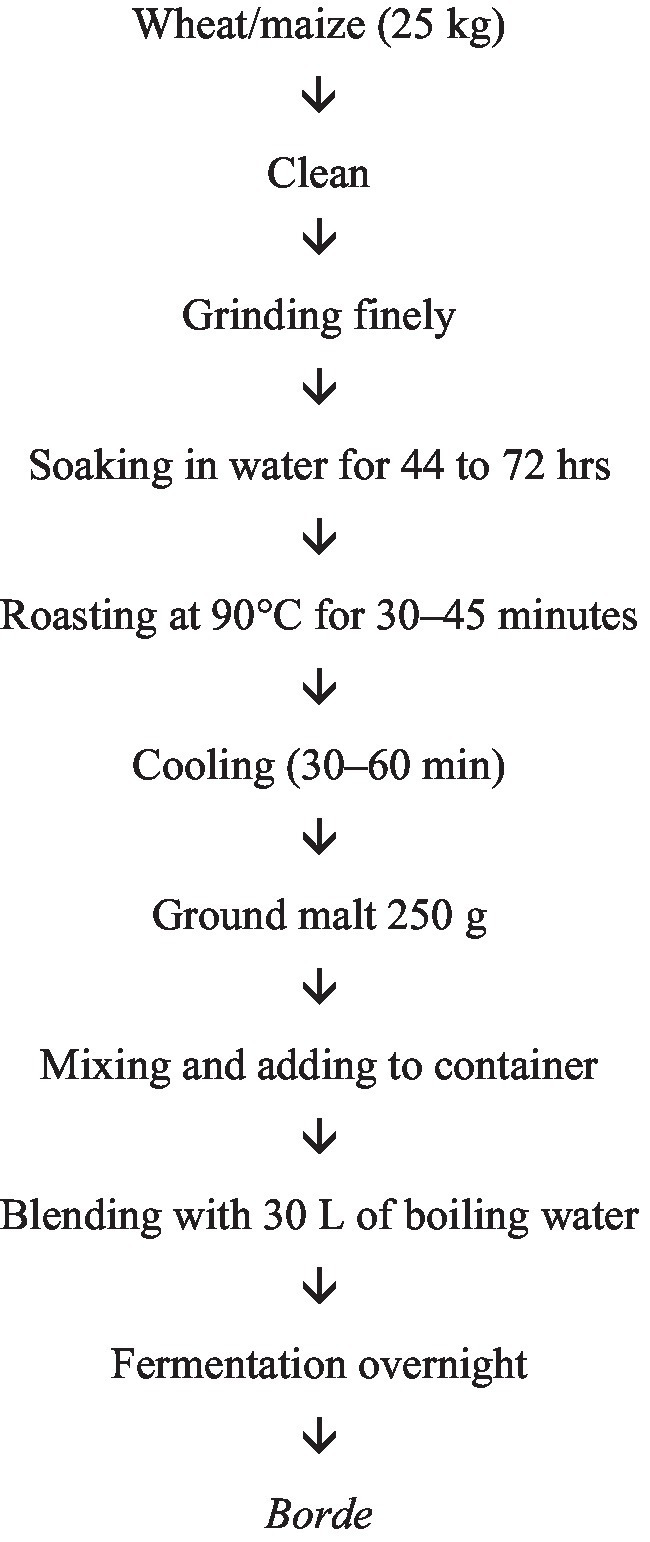
Flow chart of *Borde* fermentation.

Barley is important for the preparation of malt. However, the processing steps are not markedly different. For malt preparation, barley is cleansed to remove dirt and extraneous materials and is steeped in water for approximately one day. Excess water is drained, and the soaked barley is allowed to germinate for five days, after which it is wrapped in banana leaves. Later, germinated barley can be sun-dried and ground finely (11) (Figure 3).

#### 2.3.2 Microbiology of *Borde* fermentation

A previous study by Ashenaf (28) on the microbiological and nutritional properties of ready-to-consume *borde* in Awassa town reported that the mean pH value of the *borde* was reduced to 4.1 and that the counts of aerobic mesophilic bacteria and lactic acid bacteria were high (approximately 10^9^ CFU/mL). The counts of Enterobacteriaceae were approximately 10^6^ CFU/mL, whereas the yeast count ranged between 10^7^ CFU/mL and 10^8^ CFU/mL for the product. Lactic acid bacteria had initial counts of 10^5^ CFU/mL and reached counts as high as 10^9^ CFU/mL after 24 h. Hetero fermentative lactobacilli dominated the lactic flora throughout fermentation, and a steady increase in the yeast count was observed as fermentation proceeded (25). During fermentation, the early hours of fermentation are dominated by AMB, Staphylococci, Enterobacteriaceae, and ASFB. However, at the end of fermentation, LAB and yeast dominated the fermentation and reached maximum counts of 7.33 ± 0.07 and 6.91 ± 0.04, respectively. Microbes such as *Streptococcus*, *Bacillus*, and *Corynebacterium* started fermentation, which was later dominated by lactic acid bacteria (LAB) and yeast. In a microbial challenge test of a *borde* sample, the counts of all pathogens increased significantly until the end of the challenge test. However, the rate of growth varied: the counts of *E. coli* (3.15 ± 0.03 to 5.82 ± 0.08 log CFU/mL) and *S. aureus* (3.72 ± 0.05 to 6.05 log CFU/mL) increased by more than 2.3 log CFU/mL, whereas those of *S. typhimurium* (3.48 ± 0.04 to 5.44 ± 0.01 log CFU/mL) and *Candida albicans* (3.57 ± 0.07 to 5.09 ± 0.09 log CFU/mL) increased by less than 2 log CFU/mL (7). All pathogens reach the infective dose in *borde* beverages. *Borde* supported the growth of pathogens because of its low alcoholic content, low acidity (4.22) due to the short fermentation time of the beverages (overnry fermentation), and good nutritional profile for the proliferation of microorganisms (26) (Table 3).

**Table 3:** Microbial dynamics (means ± SDs) during fermentation of *Borde*.

Fermentation time (h)	Mean microbial count (log CFU/mL)						
	AMB	Staphylococci	Enterobacteriaceae	Coliform	ASFB	LAB	Yeast
0	6.42 ± 0.10	5.44 ± 0.08	5.40 ± 0.11	3.35 ± 0.07	4.45 ± 0.06	4.75 ± 0.04	4.12 ± 0.16
6	6.15 ± 0.05	5.29 ± 0.06	4.17 ± 0.07	2.60 ± 0.11	4.26 ± 0.04	5.34 ± 0.01	4.45 ± 0.30
12	5.20 ± 0.02	4.87 ± 0.01	3.85 ± 0.09	< 2	4.04 ± 0.06	5.55 ± 0.14	5.22 ± 0.09
18	5.10 ± 0.01	4.15 ± 0.05	< 2	< 2	3.98 ± 0.05	6.09 ± 0.04	5.86 ± 0.06
24	4.99 ± 0.06	3.85 ± 0.09	< 2	< 2	3.77 ± 0.11	7.33 ± 0.07	± 0.04

Source: Nemo and Bacha (7).

### 2.4 Shamita

*Shamita* is a traditional fermented beverage widely consumed in different regions of Ethiopia, especially by the *Gurage* people. It is low in alcohol content and is made by overnight fermentation of mainly roasted barley flour. Because of its thick consistency and ability to be a good source of protein, *shamita* is known to be used as a meal replacement. However, it has poor quality and must be consumed within a few hours after being ready for consumption (8). *Shamita* is a locally produced and consumed porridge that is used mainly to support the strength and recovery of lactating women after birth (10). *Shamita* plays a significant role in Ethiopia, particularly among the *Gurage* people and other communities where it is traditionally consumed. This fermented beverage not only is a staple in the diet but also plays vital roles in various social and cultural contexts. In Ethiopian culture, *shamita* is consumed during various social gatherings and ceremonies, such as weddings, naming ceremonies, and festivals. It serves not only as a beverage but also as a medium for social interaction and bonding among community members (29).

#### 2.4.1 Preparation of *Shamita* fermentation

**Figure 4:**
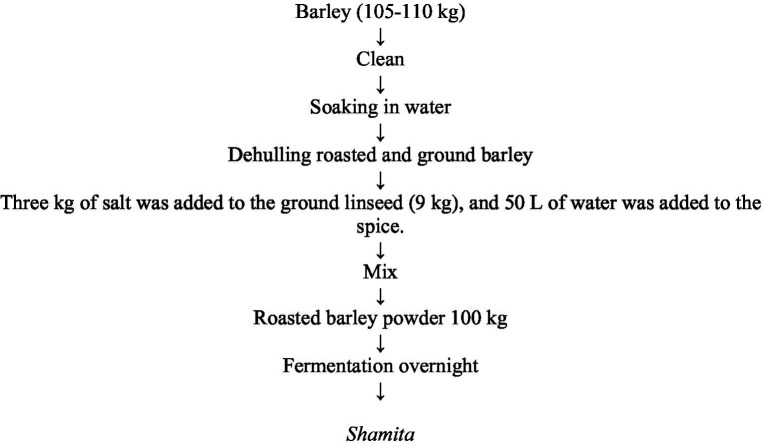
Flow chart of *Shamita* beverage preparation.

*Shamita* is typically prepared from maize and wheat. The availability of soluble protein initially increases but then gradually decreases during the preparation process. Often, *shamita* is made by grinding roasted barley and mixing it with salt, ground linseed, and spices to enhance its flavor. Unlike other varieties, *shamita* is a fermented porridge with differences in fermentation time, preparation methods, utilization and ingredient composition (4). The preparation process of *shamita* starts by mixing 2 liters of water with little flour. Bake the dough into bread and shred the bread into smaller pieces. Then, a clean container was prepared, and the pieces of bread were mixed with minimal malt. Three more liters of water were added and mixed thoroughly. After that, the container was covered, and the mixture was allowed to sit for 3 days, after which it was fermented. On the third day, 7 more liters of water were poured. The mixture was then strained via a sieve into another clean container. The container was covered and allowed to sit for approximately 2 h. In the meantime, a large bowl was used to add the *besso* to the first mixture. Two liters of water were then added, and the mixture was mixed thoroughly. Next, this mixture was added to the main mixture, and 3 more liters of water were added to the spices. The samples were mixed thoroughly and covered. Finally, the mixture was filtered into a new container. You now have your *shamita* ready to serve and enjoy (29) (Figure 4).

#### 2.4.2 Microbiology of *Shamita* fermentation

Bacha et al., studied *Shamita* fermentation microbial dynamics and the microbial load of raw materials. Their study revealed that barley is the major source of fermentative microorganisms. The count of these fermentative microbes reached 10^9^ CFU/mL after a 24-h fermentation period (29). Later (30), studied the antimicrobial effect of LAB isolated from *shamita* on pathogenic microorganisms. The isolated LAB were found to inhibit the growth of the *Salmonella* species *S. flexneri* and *S. aureus* (31). LAB are the predominant microorganisms during *shamita* fermentation, with significant contributions from both homofermentative and heterofermentative *Lactobacillus* species. Its count can reach 10^9^ CFU/mL by the end of fermentation, playing a crucial role in acidifying the product and enhancing its flavor and safety by inhibiting spoilage organisms and pathogens. Various *Bacillus* species, including *B. subtilis*, *B. licheniformis*, *B. megaterium*, *B. coagulans,* and *B. circulans,* are also commonly associated with *shamita* fermentation. These aerobic mesophilic bacteria contribute to the overall microbial profile and can be found in high numbers, particularly in the initial phases of fermentation (32). In addition to LAB and *Bacillus* species, other aerobic mesophilic bacteria are present, contributing to fermentation dynamics. Their levels are generally lower than those of LAB but are significant in the overall microbial community. Initially, present in fermentation substrates, *coliforms* and other members of Enterobacteriaceae are typically eliminated during fermentation due to the competitive advantage of LAB, which produce organic acids that lower the pH and create an unfavorable environment for these bacteria (29).

### 2.5 Keribo

*Keribo* is a traditional beverage in Ethiopia that is favored by those seeking low-alcohol drinks with limited financial resources (33). It is widely consumed in Ethiopia’s rural and urban areas, particularly in the southern, southwestern, and eastern regions. This beverage has a brief shelf life of 2 days when stored at room temperature (11). In Ethiopia, especially in the southwestern region, the fermentation of *keribo* is intertwined with sociocultural practices, mainly due to religious reasons for people who do not consume alcoholic beverages, with *keribo* serving as a source of energy (34). It is used for household consumption and during wedding ceremonies and holidays in various parts of Ethiopia.

#### 2.5.1 Preparation of *Keribo* fermentation

**Figure 5:**
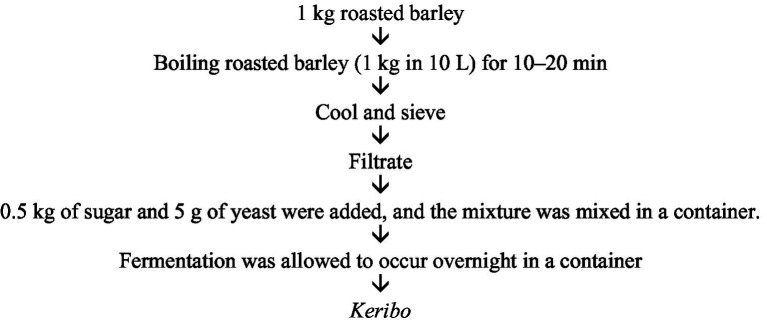
*Keribo* fermentation process.

Barley, honey, and sugar are primarily used to produce *keribo.* To make *keribo,* roasted barley is mixed with hot water. The barley grains are meticulously cleaned before being processed for *keribo* (11). The fermentation of *keribo* starts by preparing a clean container and adding 3 liters of water along with half of the wheat malt. Then, the pots were covered, and the plants were allowed to sit for 3 days. Simultaneously, in a different container, 2 liters of water were mixed with the teff flour. After 3 days, the bread was prepared from the teff flour mixture that was previously made, which was allowed to cool and then shred into small pieces. Then, the pieces of bread were added to the first mixture, which was made 3 days prior, along with 250 grams of wheat malt and 5 liters of water, and mixed well. Next, 5 kilograms of barley flour were prepared by roasting it in a separate pan until it changed color to dark brown while sprinkling it with water. The *enkuro was allowed to* cool before mixing with the remaining malt. The mixture was then added to the previous mixture along with 1 liter of water and mixed thoroughly. The mixture was covered and allowed to sit until the next day. The next day, 15 liters of water were poured into the *enkuro* mix, which was covered, and allowed to sit for 3 more days. After 3 days, filter your mixture, and you now have your *keribo* ready to serve and enjoy (35) (Figure 5).

#### 2.5.2 Microbiology of *Keribo* fermentation

A study by Abawari, examined samples of *keribo* from open markets and households in the Jimma zone. The average LAB, aerobic mesophilic bacteria (AMB), aerobic spore formers (ASFs), and yeasts had mean counts of 2.70 ± 2.07, 2.34 ± 2.37, 4.96 ± 2.80, and 4.96 ± 0.60 (log CFU/mL), respectively, after 6 h of fermentation (36). However, the microbiology of the *keribo* samples drawn at intervals during controlled laboratory fermentation was associated with mean counts of coliform bacteria, Enterobacteriaceae, Enterococci, and Staphylococci below the detection level. The initial high pH of 5.75 during *keribo* fermentation at 0 h could explain the growth of aerobic mesophilic bacteria (AMB), while the lower pH (pH = 4.47) at 6 h of fermentation inhibited their growth. The high numbers of LAB attained after 6 h of fermentation were responsible for the marked reduction in pH and increase in titrable acidity, resulting in the inhibition of most aerobic mesophilic bacteria (AMB). The mean counts of yeasts increased throughout fermentation (for 48 h) of the laboratory-prepared *keribo*. Similarly, there was an increase in the number of LAB and aerobic spore formers (18).

### 2.6 Korefe

*Korefe* is the name offer the traditional indigenous fermented beverage made in the northern and northwestern parts of Ethiopia (8). Like other fermented Ethiopian beverages, its fermentation system is natural and spontaneous. Barley, malted barley, gesho (*R. prinoides*), and water are the major ingredients used to prepare this indigenous beverage (37).

#### 2.6.1 Preparation of fermentation products from *Korefe*

**Figure 6:**
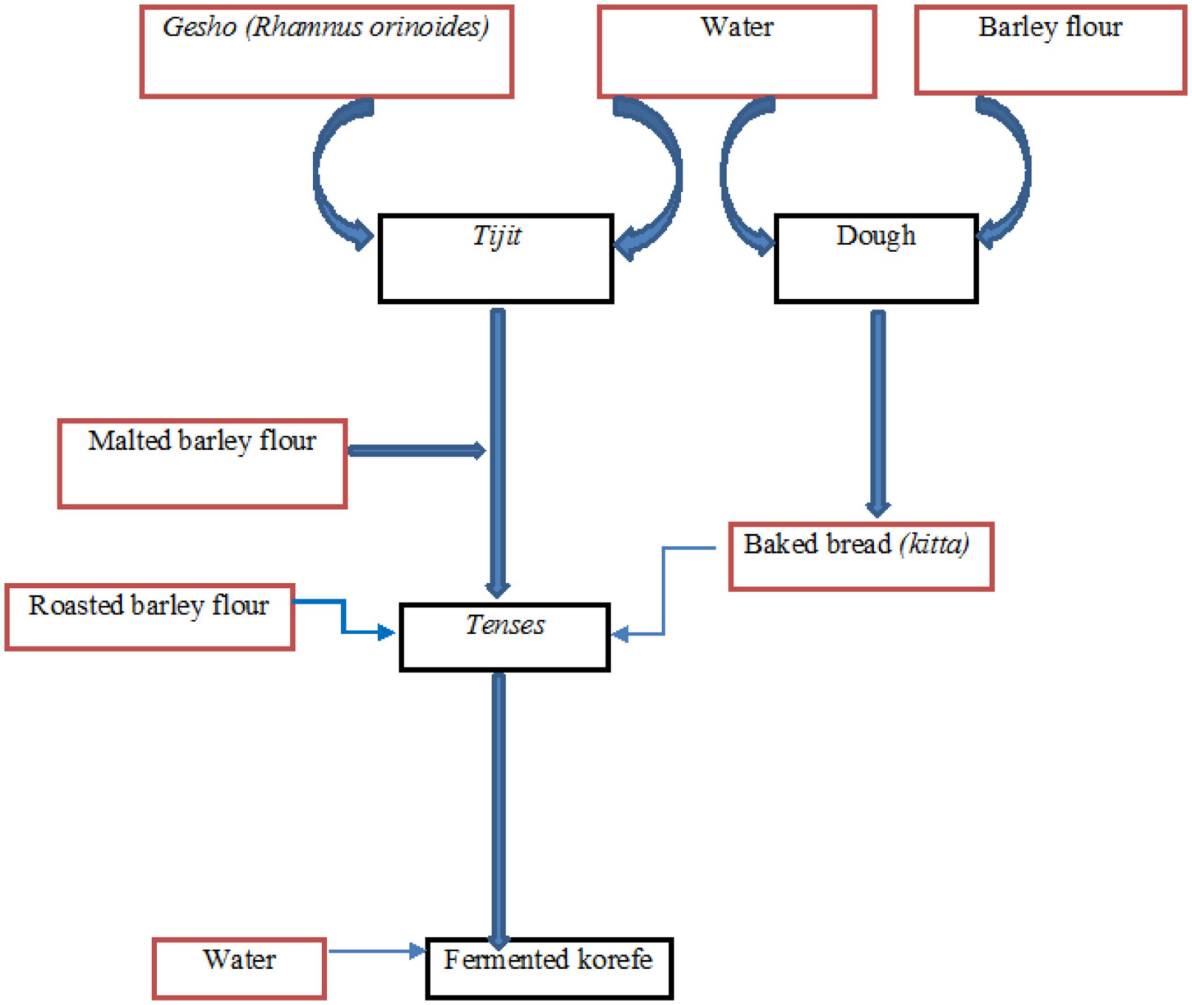
Flow chart of *Korefe* fermentation.

The first stage in the production of *korefe* is combining water and gesho (*Rhamnus prinoides*) to produce *tijit* in a traditional container called Gan. This mixture is left for 72 h to extract flavor, aroma, bitterness, and fermenting microorganisms (38). The non-malted powder is then mixed with water to form dough, which is then baked into unleavened bread called *kitta. Tijit*, a small mixture of *kitta* and water is mixed and left to ferment for approximately 48 h, resulting in a semisolid mixture called *tenses* (18). At this stage, the non-malted roasted barley powder (*derekot*) is added to the *tenses* and allowed to ferment for an additional 72 h. Finally, water was added to the mixture at a ratio of 1:3. After 2–3 h of fermentation, the *korefe* is ready to drink (38) (Figure 6).

#### 2.6.2 Microbiology of *Korefe* fermentation

According to (38), the colony count of microorganisms during fermentation varies between 3.6 log CFU/mL and 9.7 log CFU/mL. The numbers of Lactococci and Lactobacilli increased from 4 log CFU/mL to 9.7 log CFU/mL during fermentation. Yeasts are organisms that are responsible for fermentation. The bacterial count increased from 3.6 log CFU/mL to 9.3 log CFU/mL, whereas the Enterobacteriaceae count was below the detectable limit because of the antagonistic effect of lactic acid bacteria. In addition, a study by Hirbo et al., on the effects of microbial activity on fermented *korefe* for 1 month *revealed* 0.11 ± 0.21 CFU/mL *E. coli*, 0.34 × 10^3^ CFU/mL Enterobacteriaceae, 0.22 × 10^3^ CFU/mL *Lactobacillus*, and 0.02 × 10^3^ CFU/mL coliforms (39).

### 2.7 Tej

*Tej* is an Ethiopian traditional alcoholic beverage with significant social and economic importance. It is one of the most popular traditional alcoholic beverages in the country. *Tej* is typically household commercial products sold for consumption at the point of production (12). The traditional fermented beverage *tej* is yellow in color, has a sweet taste, is used to satisfy thirst, and has medicinal value similar to traditional methods (7).

Ethiopia has the potential to produce 500,000 tons of bee honey annually. However, production has not surpassed 10% of that potential (40). Approximately 80% of the total honey produced in the country serves as raw material for producing *tej* (41). Traditionally, crude honey rather than refined honey is preferred for the production of *tej* due to the distinct sensorial properties that local consumers prefer (42).

#### 2.7.1 Preparation of *Tej* fermentation

**Figure 7:**
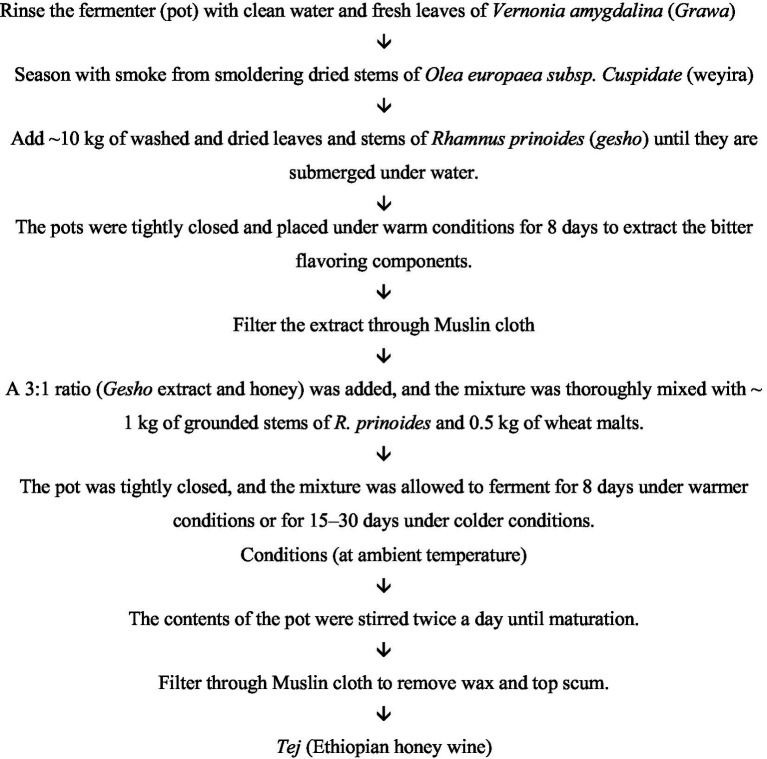
Flow chart for the preparation of traditional *Tej.*

Crude honey and *gesho* (*Rhamnus prinoides*) are the basic ingredients of high-quality *tej* (8). The process begins by mixing honey and water in a 1:3 ratio. After 2–3 days of primary fermentation, the blend was subsequently filtered through a clean cheesecloth. Then, boiled *gesho* leaves and stems are added to the honey water filtrate. At this point, some communities use fresh *gesho* leaves instead of dried and boiled leaves and stems. Furthermore, some producers add small amounts of malt powder to boiled *gesho* leaves (7). Regardless of the ingredients, the blend underwent secondary fermentation for 8–21 days. Finally, the mixture was filtered and served as the final product, *tej* (Figure 7).

#### 2.7.2 Microbiology of *Tej* fermentation

The microorganisms involved in the fermentation process originate from the raw materials, equipment and utensils. Because of this, *tej* fermentation is lengthy, spontaneous, and uncontrolled. Thus, the final product has inconsistent physicochemical properties, microbiological profiles, and sensory attributes (26). In *tej* fermentation, the counts of AMB until 36 h and *staphylococci* and ASFB until 24 h increased by more than 1 log CFU/mL. However, the count of yeast increased by more than 5 logs (4.16 ± 0.04 to 9.41 ± 0.06 log CFU/mL), and the count of LAB increased by nearly 5 logs (4.01 ± 0.03 to 8.88 ± 0.01 log CFU/mL), with a significant difference in counts between fermentation hours analyzed from the beginning (0 h) to the end of fermentation (144 h) (7). This value was much greater than that reported by Nemo and Bacha (26), who reported values of 6.31 ± 0.63 and 6.09 ± 0.53 log CFU/mL from Jimma town *tej* vendors. In *tej* fermentation, the highest counts of yeast and LAB coexisted, originating from the raw materials: honey, malt, and hops. A report on the growth potential of pathogens in the *tej* sample revealed that *E. coli* and *S. typhimurium* had greater survival abilities than did *S. aureus* and *L. monocytogenes*. Moreover, *C. albicans* was reduced to a lesser extent (0.88 CFU/mL) than the other pathogens were. However, *L. monocytogenes* was highly reduced (2.2 CFU/mL), with a significant difference among counts (7) (Table 4).

**Table 4:** Microbial dynamics (means ± SD) during fermentation of fermented *tej.*

Fermentation time (h)	Mean microbial count (CFU/mL)						
	AMB	Staphylococci	Enterobacteriaceae	Coliform	ASFB	LAB	Yeast
0	5.37 ± 0.04	3.90 ± 0.02	4.34 ± 0.07	3.23 ± 0.02	4.13 ± 0.02	4.01 ± 0.03	4.16 ± 0.04
12	5.94 ± 0.04	4.73 ± 0.01	4.70 ± 0.06	3.55 ± 0.01	4.25 ± 0.01	4.83 ± 0.06	4.97 ± 0.04
24	6.58 ± 0.02	5.63 ± 0.11	5.17 ± 0.07	3.51 ± 0.08	5.42 ± 0.01	5.53 ± 0.13	5.39 ± 0.07
36	6.62 ± 0.01	4.41 ± 0.03	4.26 ± 0.04	3.38 ± 0.07	4.36 ± 0.03	5.94 ± 0.03	6.62 ± 0.07
48	6.36 ± 0.16	4.31 ± 0.02	3.82 ± 0.06	3.15 ± 0.04	4.17 ± 0.10	6.60 ± 0.08	6.77 ± 0.01
60	5.41 ± 0.09	4.05 ± 0.08	3.28 ± 0.08	2.83 ± 0.03	3.83 ± 0.06	6.65 ± 0.04	6.84 ± 0.06
72	5.29 ± 0.08	3.93 ± 0.02	3.13 ± 0.01	< 2	3.21 ± 0.01	7.10 ± 0.03	7.40 ± 0.05
84	4.60 ± 0.10	3.22 ± 0.02	3.01 ± 0.04	< 2	3.14 ± 0.01	7.51 ± 0.07	7.53 ± 0.04
96	4.28 ± 0.21	2.77 ± 0.01	2.55 ± 0.17	< 2	2.83 ± 0.05	7.84 ± 0.07	8.43 ± 0.06
108	3.64 ± 0.04	2.55 ± 0.01	< 2	< 2	2.69 ± 0.07	8.15 ± 0.06	8.61 ± 0.07
120	3.52 ± 0.04	2.34 ± 0.02	< 2	< 2	2.51 ± 0.07	8.41 ± 0.08	8.76 ± 0.11
132	3.45 ± 0.03	2.13 ± 0.01	< 2	< 2	2.34 ± 0.01	8.62 ± 0.08	9.01 ± 0.04
144	3.42 ± 0.01	2.07 ± 0.06	< 2	< 2	2.22 ± 0.02	8.88 ± 0.01	9.41 ± 0.06

Source: Nemo and Bacha (7).

### 2.8 Booka

*Booka* is an indigenous traditional fermented beverage in South Ethiopia that is particularly consumed in *Guji* communities. It is the first animal-origin traditional fermented beverage in which a slightly yellowish liquid is made of *booka* from cow bladders, so the product is named *booka*. *Booka* is sometimes found at the bottom of *buttee* (a traditional instrument that is used to ferment milk). However, the beverage that is used to ferment the beverage (*booka*) is usually cow bladder. People of all ages, including infants, pregnant, and lactating women, drink bookas (13). It has been consumed for ceremonies such as marriage, blessing (*Eebbaa*), *Gadaa* power transition (*Baallii dabarsaa*), and conflict resolution (*Araara*) and as a source of income generation.

#### 2.8.1 Preparation of *Booka* fermentation

**Figure 8:**
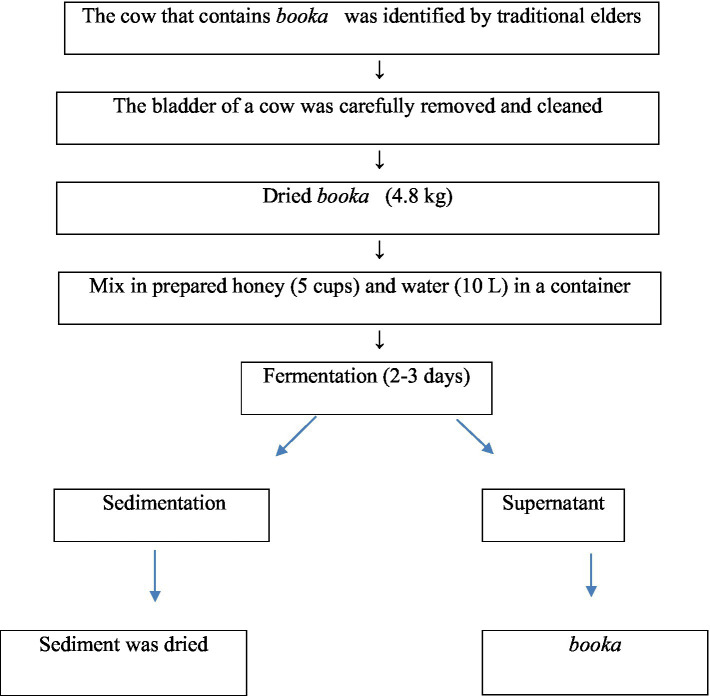
Flow chart of *Booka* preparation.

The indigenous production and preparation methods of bookas are very simple; they can be prepared using equipment such as a wooden bowl (*Qorii*), cup (*Kookkii*), container (*gan*), or filter. In contrast, ingredients such as honey, sugar (sometimes), water and *booka are* from certain types of cow bladders. The *Guji* people (mostly older people) know which cows to select for this process, but the exact cause of the existence of the *booka* in the bladder of a cow is unknown. The liquid from the bladder of a cow is carefully removed, cleaned, and then filled with honey and water in a container. This mixture is subsequently enhanced with pure honey and used immediately because the fertility of this *booka* will increase if this type of pure honey is used. However, if they need to preserve for a long period, they mix fresh *booka* with honey and water at an appropriate ratio and then dry, pack, and put for future usage. For immediate use, they inoculated this active *booka* in honey and water in a container and stored it for two to three days, during which time it underwent fermentation. After fermentation is complete, the upper layer is then ready to drink, while the bottom layer (sediment) is reused again to ferment another *booka* (beverage). The good-quality *booka* is yellowish in color, sweet in taste, and attractive in odor. The popularity of this traditional fermented beverage is greater among all age groups (13) (Figure 8).

#### 2.8.2 Microbiology of *Booka* fermentation

The microbial activities of *E. coli,* Enterobacteriaceae, *Lactobacillus* and *coliform bacteria* in *booka* were 0.51, 1.67 × 10^1^, 1.2 × 10^4^ and 3.67 × 10^5^, respectively_._ The effects of microbial activity on fermented *booka* during the first month of the study included *E. coli* 0.41 ± 0.20 (CFU/mL), Enterobacteriaceae 0.13 × 10^2^ (CFU/mL), *Lactobacillus* 0.07 × 10^3^ (CFU/mL), and coliforms 1.67 × 10^1^ (CFU/mL) (39).

### 2.9 Cheka

*Cheka* is a traditional fermented beverage primarily consumed in southwestern Ethiopia, especially in the Dirashe and Konso districts. This beverage is made from a mixture of cereals and vegetables, and it plays a significant role in local culture and diet (43). *Cheka* is not just a beverage but also a cultural staple consumed by people of all ages, including infants and pregnant women. It is often seen as a low-cost meal alternative, particularly for low-income individuals. The beverage is consumed throughout the day, with some adults reportedly drinking up to 8 liters daily. It is commonly served during social gatherings and special occasions, reinforcing its role in community bonding and cultural practices (44).

#### 2.9.1 Preparation of *Cheka* fermentation

**Figure 9:**
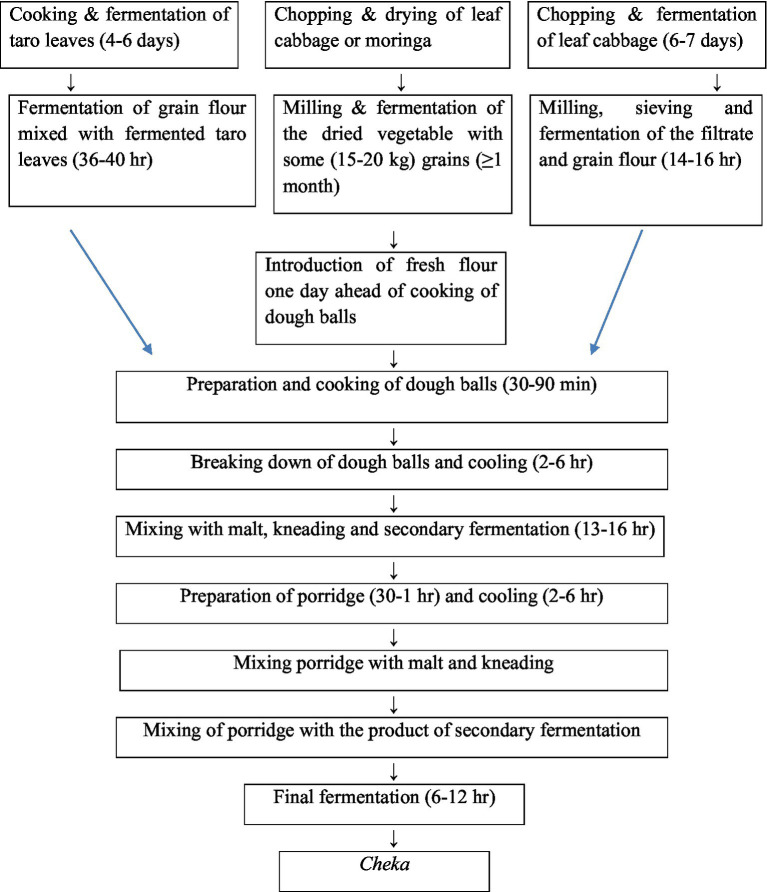
Flow chart for *Cheka* preparation.

*Cheka* is mainly prepared from cereals such as sorghum (*Sorghum bicolor*) and maize (*Zea mays*) and vegetables such as leaf cabbage (*Brassica* spp.), moringa (*Moringa stenoptella*), and decne (*Leptadenia hastata*). Additionally, the root and leaf parts of the taro were used. The processes of *Cheka* preparation are very complex and vary among households and localities. There are three types of *Cheka* produced in Konso and Dirashe: *hiba, chaqa*, and *menna* (45). The study by Worku et al. (46) reported a survey of raw materials and the production process of *Cheka*. According to their report, *Cheka* preparation starts with malting. The malt is prepared from either a single or a combination of the cereals listed above. Cabbage leaves and/or taro roots are cut into pieces and fermented anaerobically for approximately 4 to 6 d in a clean container. Then, a small amount of maize flour is added to the vegetable mixture, which is fermented for an additional 2 to 3 days. The fermented vegetable mixture was then ground, filtered, and mixed with fresh maize flour. The fermentation continues for another 12 to 24 h. Then, water was added to the mixture, and the mixture was allowed to ferment for one month. This fermented mixture is shaped into a dough ball, locally called *gafuma,* and cooked at a temperature of 96°C. After cooling, the cooked *gafuma* is mixed with an adequate amount of previously prepared malt. The mixture was then allowed to ferment for an extra 12 h. This fermented mixture is locally called *sokatet*. At this stage of the process, a very thick porridge, locally called *koldhumat*, is prepared from maize flour. The prepared porridge was added to a vessel containing a *sokatet* with a sufficient amount of water. Finally, the mixture is left to ferment for other 4 to 12 h and serves consumers as *Cheka*. It has a shelf-life of 2–4 days. However, it is usually produced on a small-scale basis to avoid loss. *Cheka* is ready for consumption after 4–12 h of fermentation. As the duration of fermentation in the preparation of *hiba* (Dirashe *Cheka*) is too long, the *sokatet* becomes much bitter, and as a result, the amount of malt added into *hanshalt* in the preparation of fasha (Konso *Cheka*) is slightly greater than that added to *hiba*, and the proportion of *sokatet* in the final product is much greater than that of *hanshalt* in *fasha* (Figure 9).

#### 2.9.2 Microbiology of *Cheka* fermentation

*Cheka,* a traditional fermented beverage from Ethiopia, is produced through the action of various microorganisms during fermentation. The main types of microorganisms involved in the fermentation of *Cheka* include *Lactobacillus* spp., which are crucial for fermentation, contributing to sour flavor, preserving, lowering pH, and enhancing beverage safety and stability*. Additionally, Saccharomyces cerevisiae* is involved in converting sugar present in cereal ingredients into ethanol and carbon dioxide, which contribute to the alcohol content of beverages. The fermentation process is typically spontaneous and can involve a mixed culture of various other bacteria and yeast, which may vary depending on local practices and environmental conditions (44). The presence of these microorganisms not only contributes to the organoleptic properties (taste, aroma, etc.) of *Cheka* but also affects its nutritional value and safety. The fermentation process can increase the bioavailability of nutrients and may also introduce probiotic properties that are beneficial for gut health (11). However, the uncontrolled nature of traditional fermentation can also lead to contamination, including the presence of harmful substances such as aflatoxins, which are produced by certain molds that can grow on the cereal ingredients used in *Cheka* (47).

### 2.10 Grawa

The *grawa* is a traditional Ethiopian beverage particularly popular in the Qellem Wollega Zone, Anfilo district. This beverage has not been extensively documented in the literature, making it a lesser-known aspect of Ethiopian culinary traditions than other drinks, such as *tella* or *tej*. *Grawas* are consumed during social gatherings or special occasions, reflecting their cultural significance (26). Its production is often a household practice, reflecting the traditional methods of beverage-making in Ethiopian culture. It is yellow, has a sweet taste, is used to quench thirst and has medicinal value similar to that of *tej*. It is a fermented beverage prepared from specific honey made from flowers of *Vernonia amygdalina* and water from Anfilo District. *Grawa* from previous fermentation methods (back slope) has been used as a starter culture, and the whole fermentation process takes approximately 72 h (7).

#### 2.10.1 Preparation of *Grawa* fermentation

**Figure 10:**
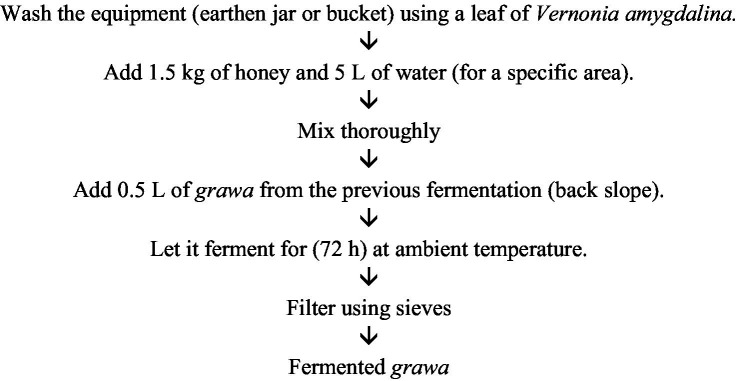
Flow chart of the *Grawa* preparation process.

Smoking the clay container used for preparing *grawa*, a traditional Ethiopian honey-based beverage, contributes to the flavor profile. The first step in the preparation of *grawa* is mixing honey with water in appropriate proportions. The exact ratio can vary on the basis of local preferences and desired sweetness. The mixture was then allowed to ferment naturally. This process typically relies on wild yeasts and bacteria present in the environment, which can vary by location. Traditionally, fermentation containers may be washed with fresh leaves of *grawa* (*vernonia amygdalina*) to impart flavor and potentially introduce beneficial microorganisms. The fermentation process was monitored over several days. The duration can vary, but it usually lasts until the desired level of fermentation is achieved, which is indicated by the development of carbonation and a slight alcoholic content. Once fermentation is complete, *grawa* is ready to be consumed (26) (Figure 10).

#### 2.10.2 Microbiology of *Grawa* fermentation

The fermentation of *grawa* typically involves a mix of microorganisms, primarily yeasts and lactic acid bacteria, which contribute to the flavor, aroma, and alcoholic content of the beverage. The spontaneous fermentation process allows for a diverse microbial community, which can influence the final characteristics of *grawa* (26). The microbial dynamics in *grawa* beverages at 0 h were greater (5.06 ± 0.02 log CFU/mL) than those of other microorganisms. The dominance of AMB continued until 24 h, and after 24 h, yeast and LAB dominated the fermentation process until the end of fermentation (72 h) and reached maximum counts of 7.88 ± 0.02 and 7.64 ± 0.04 log CFU/mL, respectively (7). A previous study by Nemo and Bacha (26) reported 8.43 ± 0.72 log CFU/mL of yeast and 8.13 ± 0.67 log CFU/mL of LAB from the *grawa* beverage collected from Anfilo District of the Qellem Wollega Zone, Southwest Ethiopia. The lower count of yeast and LAB in the present study could be due to environmental factors. In the study by Nemo and Bacha (7) in the *grawa* sample, a fast reduction (0.52 log) in *L. monocytogenes* was observed from the initial 0 h (3.90 ± 0.02 CFU/mL) to 6 h (3.38 ± 0.03 log CFU/mL), with a significant difference (*p* < 0.05) between counts, whereas *S. typhimurium* and *E. coli* were reduced by 0.1 log, with no significant difference (*p* > 0.05) between counts of different sampling hours. At the end of microbial challenge testing, *E. coli*, *S. typhimurium*, *S. aureus*, and *L. monocytogenes* were reduced by >1 log unit. However, *C. albicans* was reduced by 0.7 logs (Table 5).

**Table 5:** Microbial dynamics (means ± SD) during fermentation in *Grawa.*

Fermentation time (h)	Microbial mean count (CFU/mL)						
	AMB	*staphylococci*	Enterobacteriaceae	Coliform	ASFB	LAB	Yeast
0	5.06 ± 0.02	4.91 ± 0.06	4.29 ± 0.06	4.13 ± 0.03	3.89 ± 0.06	4.63 ± 0.03	4.73 ± 0.02
12	6.30 ± 0.12	5.14 ± 0.01	5.16 ± 0.04	4.11 ± 0.03	4.56 ± 0.14	5.70 ± 0.02	5.40 ± 0.08
24	6.41 ± 0.04	5.08 ± 0.09	4.73 ± 0.06	4.08 ± 0.06	4.51 ± 0.01	6.26 ± 0.09	6.06 ± 0.08
36	5.54 ± 0.05	4.37 ± 0.04	3.61 ± 0.08	3.03 ± 0.02	4.49 ± 0.01	6.49 ± 0.03	6.39 ± 0.06
48	4.73 ± 0.14	3.95 ± 0.14	2.80 ± 0.02	2.21 ± 0.13	3.39 ± 0.08	6.45 ± 0.01	6.67 ± 0.16
60	3.82 ± 0.05	3.62 ± 0.07	< 2	< 2	3.06 ± 0.08	7.46 ± 0.09	7.71 ± 0.09
72	3.39 ± 0.02	2.77 ± 0.01	< 2	< 2	2.58 ± 0.03	7.64 ± 0.04	± 0.02

Source: Nemo and Bacha (7).

## 3 Nutritional value and bioavailability of fermented beverages

### 3.1 Bioavailability of nutrients

Ethiopia has a rich tradition of producing and consuming a variety of traditional fermented beverages, which are integral to local food culture and nutrition. These beverages, including *Korefe*, *Booka*, *Tella*, *Shamita*, *Borde*, *Keribo*, and *Areki*, are typically made from locally sourced raw materials and reflect regional cultural practices. Apart from their cultural significance, these drinks are also valued for their nutritional benefits, such as providing essential nutrients and improving food security in rural areas. The fermentation process itself may contribute to the development of beneficial microorganisms, enhancing the nutritional value and digestibility of the drinks (4). Although the traditional fermented beverages produced in uncontrolled way of microbiota, they are sources of amino acids, antioxidant compounds, bioactive beeps, short chain fatty acids, vitamins, and minerals (48). Furthermore, a beverage made from oat blend increases the content of fat, carbohydrate, gross energy, and mineral contents (Fe, Zn) (49). It also increases the digestibility and bioavailability of different nutrients (50). The nature of beverage preparation in Ethiopia, traditional household processing, associated microorganisms with a fermented beverage, and their contribution toward improving the nutritional value and safety, the extent, and its prospect in supporting the livelihood of people in Ethiopia need concern (11). The diverse microbial species involved in beverage products result in distinct aromatic and flavor profiles (51) and extend the shelf life of the food (52). In some of the traditionally fermented beverages, Ethiopians use “*gesho”* (*Rhamnus prinoides*) providing unique characteristics of flavor (16). Moreover, traditionally fermented beverages and condiments are rich in different essential vitamins, minerals, enzymes, and antioxidants, which are enhanced through the process of traditional fermentation (8).

#### 3.1.1 Nutritional aspects of beverages

Fermentation is a traditional food processing method that produces safe and nutritious foods, although the nutritional composition and health benefits of many such foods have not been extensively studied. One example is “*Tella*,” a locally prepared beer made using ingredients like “gesho” (*Rhamnus prinoides*) and malt. The antioxidants in “gesho” have been shown to have potential health benefits, including improving bioavailability, preventing metabolic syndrome-related diseases, and offering anti-cancer effects (53). On the other hand, beverages are not usually consumed for their food value, particularly the fruit drinks, contain quite a high percentage of sugar, vitamins and minerals, and, therefore, add to the energy content of the diet (54). Study conducted in Jimma, Ethiopia confirmed that *Tella* contains crude protein content (16.47–18.72%), crude fat content (3.73–5.43%), crude fiber content (15.52–19.73%), total ash content (3.58–4.47%), and carbohydrate content (35.02–40.50%) (55). *Checka*, a traditional beverage from cereals and vegetables in Southwestern Ethiopia, provides essential nutrients such as crude protein (3.12–4.44 g/100 g), crude fat (1.17–1.81 g/100 g), crude fiber (0.65–0.93 g/100 g) and carbohydrates (82.04–107.17Kcal) (44) and it is the source of probiotics like *Weissella paramesenteroides* and *Leuconostoc pseudomesenteroides* isolates, which showed promising probiotic properties (56). On the other hand, there are various locally prepared beverages such as *Keribo*, *Borde*, *Areki*, *Tella*, *Shamita*, *Booka*, *Korefe*, and *Cheka*, which are naturally fermented. These beverages rely on lactic acid bacteria and yeast as the dominant microbes. These microbes serve as probiotics, enhancing the organoleptic properties, nutritional quality, and providing biopreservative benefits (11). While beverages are not intended to replace foods, they play an important role in providing nutrients and phytonutrients, especially phenolic acids and flavonoids. However, they are not sufficient on their own to meet requirements for vitamin E, dietary fiber, or essential fatty acids (57). The “*Grawa”* is a traditional Ethiopian beverage particularly popular in the Qellem Wollega Zone. The leaf of the plant is used to clean “*Tella*” containers, enhancing its flavor, and serves as an antimicrobial agent. It contains compounds such as flavonoids, alkaloids, saponins, tannins, triterpenoids, sesquiterpene lactones, steroids, cardiac glycosides, oxalates, phytates, cyanogenic glycosides, and phenols, which contributes to its anti-cancer and anti-inflammatory properties (58). *Cheka* is also an indigenous alcoholic beverages commonly consumed in Konso, Southwestern, Ethiopia contains protein (5.97–4.95) %, carbohydrate (59.08–64.41) %, and fibre (1.2 to 1.9) %. It also contains magnesium, calcium, iron, and zinc ranging from (10.65–11.82) mg/l, (11.05–7.79) mg/l, (7.64–10.73) mg/l and (2.57–5.33) mg/l, respectively (59). *Tej* (Ethiopian honey wine) is a home-made and traditionally fermented product. The nutritional composition of *Tej* varies across regions. It contains protein (1.38) %, fat (0.47) %, and carbohydrates (3.91) % (60). In contrast, others reported *Tej* as having lipids (0.13) %, protein (0.10) %, and carbohydrates (3.02) % (61).

### 3.2 Improvement of organoleptic properties

The organoleptic properties, texture, aroma, and flavor improve through microorganisms, which makes fermented beverages tasty (62). Fermentation of *tej* depends on microorganisms (LAB and yeast), and their metabolic products contribute to acidity and enhance the distinctive flavor and aroma of the fermentation material (31). LAB isolated from various fermented foods produces organic acids and diverse antimicrobial agents, which are responsible for maintaining the quality and sweetness of fermented foods. Yeasts of the genus Saccharomyces were reported to be responsible for the conversion of sugars to ethanol in *tej*.

A previous study by Debela et al. (63) revealed that after 10 days of fermentation, *tella* becomes more acidic because of the growth of *Acetobacter* spp., which converts ethanol to acetic acid under anaerobic conditions. The organoleptic properties of fermented beverages make them more important since they have broader acceptance (64).

### 3.3 Bio preservation properties of fermented beverages

Microorganisms contribute to bio preservation by producing natural antimicrobials to extend the shelf-life of food products. Many bacteria involved in the fermentation of foods produce bioactive molecules such as hydrogen peroxide, organic acids, and bacteriocins, which act as effective bio preservatives (65). The acid content of alcoholic beverages plays a crucial role in maintaining freshness and extending shelf-life by inhibiting the growth of pathogenic microbes. LAB for example, displays antifungal properties, contributing to the prolonged shelf-life of fermented foods by preventing spoilage. This bio preservation helps ensure that fermented foods maintain quality over time.

As fermentation continues, from a fermentation dynamics point of view, only a limited number of microorganisms resist the adverse environmental effects of the growth medium. Thus, the microorganisms that do not survive in the new environment will be lysed and become a source of protein for cell maintenance for the surviving species. This analysis works even better in natural, spontaneous and uncontrolled fermentation systems. Hence, this competition in turn decreases the nutritional value of beverages while increasing secondary metabolites such as ethanol (66).

### 3.4 Nutrient enhancement

Fermentation processes increase the digestibility and availability of nutrients (11). Enzymes such as amylases, proteases, lipases, and phytates modify primary food products through the hydrolysis of polysaccharides, phytates, proteins, and lipids (64). For example, those beverages that use malt are known to contain much more free amino nitrogen than the original grain, i.e., the partial degradation of reserve proteins in cereals makes free amino nitrogen available (67). The number of proteins and the content of water-soluble vitamins increase, whereas antinutrient factors (ANFs) in foods decrease during fermentation (62).

Lactic acid fermentation of cereals has been used as a strategy to decrease the content of anti-nutrients, such as phytate and tannins (68). This leads to increased bioavailability of micronutrients such as zinc, calcium, phosphorous, iron, and amino acids. The high microbial loads of yeast and lactic acid bacteria qualify as good sources of microbial protein (69). The mean crude protein content of fermented food increased from 0.74 to 4.58% (3-fold increase) after 48 h of fermentation (18). The highest levels of crude protein were also observed in fermentation samples enriched with 1.5 mL of *Saccharomyces cerevisiae* for 48 h (70).

## 4 Health benefits of fermented beverages

The diverse functional microorganisms in fermented foods and beverages include bacteria, yeasts, and fungi. The most remarkable aspects are their biological functions and enhanced health benefits due to the functional microorganisms associated with them. The possible health benefits of fermented beverages include the prevention of cardiovascular disease, cancer, hepatic disease, gastrointestinal disorders, inflammatory bowel disease, hypertension, thrombosis, osteoporosis, allergic reactions, and diabetes (71). The consumption of these traditional fermented beverages with meals supports digestion and promotes the growth of beneficial bacteria. Different microorganisms (bacteria, yeast, and molds) are involved in alcoholic beverage fermentation, and these microorganisms are naturally probiotic and important for human health (72). The traditional fermented foods and beverages play an important role in their potential positive effects on human health (73). Recent research indicates that the consumption of traditional foods and beverages has positive effects on human health. These foods are particularly beneficial in addressing non-communicable diseases (NCDs), gastrointestinal issues, and immune disorders. Different findings suggest that incorporating traditional foods and beverages in to diets could help improve overall human health. They play a significant role in preventing chronic diseases, highlighting their importance in maintaining long-term health (73). Besides, the fermented foods are very important in preventing chronic disease (74). Besides, Lactic Acid Bacteria isolated from “*Borde”* showed strong potential to lower cholesterol (85–90%) (75).

Probiotics have great potential for improving nutrition, soothing intestinal disorders, improving the immune system, optimizing gut ecology, and promoting overall health because of their ability to compete with pathogens for adhesion sites, antagonize pathogens, or modulate the host’s immune response, pharmaceutical preparations, and functional foods to improve public health (76). Most probiotic products contain LAB and molds that have been found to produce antibiotics and bacteriocins. LAB belong to the genera *Lactobacillus*, *Bifidobacterium*, *Enterococcus*, *Lactococcus*, *Streptococcus*, and *Leuconostoc*; *Lactobacillus plantarum* strain; *Lactobacillus paracasei* strain; and *Lactobacillus plantarum* strain (77), which is inherently present in fermented *Borde* and *Shamita* and has antimicrobial properties against various foodborne pathogens invading the gastrointestinal tract. Thus, a better understanding of the intestinal microbial populations will contribute to the development of new strategies for the prevention and/or treatment of several diseases. However, some fermenting microorganisms isolated from “*Checka*” like, lactic acid bacteria and yeast, are used as probiotics, for improvement of organoleptic properties, for provision of nutritional quality and biopreservative methods (11).

Ethiopian locally fermented beverages result from acid–alcohol fermentation and are typically made from cereals such as barley, maize, and wheat (11). Barley, in particular, is gaining renewed interest because of its nutraceutical benefits. Its properties are known to offer protection against degenerative diseases, including diabetes, obesity, hypertension, and colon inflammation, which are often associated with unhealthy diets and lifestyles (78).

Traditional alcoholic beverages such as *tej* can be vital sources of calories as well as a source of vitamin B. The presence of vitamin B in *tej* is a result of fermenting yeasts, substrate residues and other microorganisms (79). Two ingredients in *tej* production honey and *R. prinoides* have medicinal properties. Additionally, *Rhamnus staddo*, which is sometimes used in *tej*, is being researched for its potential as an antimalarial (80) and *gesho* extract during *tella* brewing can inhibit bacteria growth and thereby help to extend the self-life of the product (81). The traditional beverage made from “*grawa”* (*Vernonia amygdalina*) helps reduce harmful microorganisms while promoting beneficial microbes, such as yeasts and lactic acid bacteria (LAB), which can contribute to human health by producing secondary metabolites (26). Various plant parts are used in traditional medicine to treat numerous health issues, such as diabetes, diarrhea, headache, malaria, gastritis, and snake bites, among others (58). These plants are known to deliver a range of bioactive compounds, acting as natural antioxidants that may support human health (82). Besides, it was reported that worldwide fermented foods are recognized as healthy and safe (83).

The fermentation process breaks down compounds in food into more easily digestible forms, making it easier for the body to process nutrients. It also helps to reduce harmful toxins and pathogens present in food, improving food safety. Fermented foods contain probiotics which are beneficial bacteria that support digestion and help the body absorb nutrients more effectively (84). The fermentation process helps to break down compounds into forms that are easier to digest. It also helps to reduce toxins and pathogens in food. Additionally, fermented foods contain probiotics, which are beneficial bacteria that help the body to digest food and absorb nutrients (84). On the other hand, traditionally fermented beverages prepared from “*Grawa*” (*Vernonia amygdalina* Del) treats different types of diseases like diarrhea, diabetes, wound healing, tonsillitis, evil eye, retained placenta, headache, eye disease, intestinal parasite, bloating, hepatitis, toothache, anthrax, malaria, urine retention, gastritis, stomach disorders, and snake bites (58, 85, 86) and has antimicrobial activity (87).

## 5 Public health risks associated with traditionally fermented beverages

Although there aren’t many reports of foodborne illnesses caused by eating traditional fermented foods, fermentation is an ancient technique that can enhance nutrient contents and sensory qualities while also possibly lowering or getting rid of harmful bacteria and natural toxins (88, 89). Though *Salmonella* sp., *Campylobacter* sp., and *Shigella* sp. are frequently found in beverages, the most frequently isolated harmful bacterial species were not only *Staphylococcus aureus*, *Bacillus cereus*, and *Vibrio parahaemolyticus* (90). Additionally, mycotoxins have been found in traditional brewed “*Cheka*” and beverages made from fermented tropical crops (91, 92). Furthermore, certain beverages are susceptible to methanol contamination (93). Others contain, toxic compounds such as biogenic amines and phenols that affect the quality of the product and human health has been detected (94).

## 6 Conclusion

The most widely produced and consumed traditional alcoholic beverages in Ethiopia are *tella*, *borde*, *shamita*, *korefe, cheka*, *keribo*, *tej*, and *booka*. These drinks vary from location to location in terms of ingredients and preparation technique. These drinks also differ from one another in terms of alcohol content. Traditional low-alcoholic beverages have higher nutritional value than high-alcoholic beverages do. As fermentation progresses, mesophilic aerobic bacteria and coliform bacteria dramatically decrease, whereas lactic acid bacteria and yeast species increase. These factors help to maintain the shelf life and quality of fermented beverages. The ingredients and preparation techniques for traditional fermented beverages have been thoroughly researched. To increase the quality and safety of these traditional beverages, more research is necessary to standardize the fermentation process and identify important microbial species.
